# SARS-CoV-2 Variant of Concern B.1.1.7: Diagnostic Sensitivity of Three Antigen-Detecting Rapid Tests

**DOI:** 10.1128/spectrum.00763-21

**Published:** 2022-01-05

**Authors:** Andreas K. Lindner, Lisa J. Krüger, Olga Nikolai, Julian A. F. Klein, Heike Rössig, Paul Schnitzler, Victor M. Corman, Terry C. Jones, Frank Tobian, Mary Gaeddert, Susen Burock, Jilian A. Sacks, Joachim Seybold, Frank P. Mockenhaupt, Claudia M. Denkinger

**Affiliations:** a Charité - Universitätsmedizin Berlin, Freie Universität Berlin and Humboldt-Universität zu Berlin, Institute of Tropical Medicine and International Health, Berlin, Germany; b Division of Clinical Tropical Medicine, Center of Infectious Diseases, Heidelberg University Hospital, Heidelberg, Germany; c Charité - Universitätsmedizin Berlin, Freie Universität Berlin and Humboldt-Universität zu Berlin, Medical Directorate, Berlin, Germany; d Department of Virology, Centre of Infectious Diseases, Heidelberg University Hospital, Heidelberg, Germany; e Charité - Universitätsmedizin Berlin, Freie Universität Berlin and Humboldt-Universität zu Berlin, Institute of Virology, Berlin, Germany; f German Centre for Infection Research, Partner Site Charité, Berlin, Germany; g Centre for Pathogen Evolution, Department of Zoology, University of Cambridge, Cambridge, United Kingdom; h Charité - Universitätsmedizin Berlin, Freie Universität Berlin and Humboldt-Universität zu Berlin, Charité Comprehensive Cancer Center, Berlin, Germany; i Foundation for Innovative New Diagnostics, Geneva, Switzerland; j German Centre for Infection Research, Partner Site Heidelberg, Heidelberg, Germany; Johns Hopkins Hospital

**Keywords:** B.1.1.7 lineage, COVID-19, SARS-CoV-2, antigen-detecting rapid test, variants of concern

## LETTER TO THE EDITOR

The severe acute respiratory syndrome coronavirus 2 (SARS-CoV-2) B.1.1.7 lineage (labeled Alpha by WHO) rapidly emerged after its first identification in the United Kingdom in late 2020 (1). As of 1 June 2021, B.1.1.7 was verified in 160 countries and had become the dominant variant in several European countries, including Germany, and in North America (2–4). In November 2021, the global epidemiology of SARS-CoV-2 is characterized by a predominance of the B.1.617.2 lineage (labeled Delta), with the prevalence of other variants continuing to decline (B.1.1.7 prevalence is <0.1%) (5). B.1.1.7 is defined by a large number of mutations in the spike (S) gene and four in the nucleocapsid (N) gene (N protein substitutions D3L, R203K, G204R, and S235F) (6). Virus mutations have the potential to impact the accuracy of diagnostic tests. Most commercially available SARS-CoV-2 antigen-detecting rapid diagnostic tests (Ag-RDTs) target the viral N protein, encoded by the N gene (7). In an analytical evaluation by Public Health England, the B.1.1.7 variant did not affect the performance of six commercially available Ag-RDTs, all of which target the N protein, despite a limited number of amino acid changes from the original viral sequence in the target antigen (6, 8).

We conducted a manufacturer-independent, prospective diagnostic accuracy study of three SARS-CoV-2 Ag-RDTs at ambulatory testing facilities in Berlin and Heidelberg, Germany, from 20 January to 15 April 2021. Two Ag-RDTs evaluated are currently under review by the WHO Emergency Use Listing Procedure, i.e., (i) Espline SARS-CoV-2 (Fujirebio Inc.), using nasopharyngeal swab sampling, and (ii) Mologic coronavirus disease 2019 (COVID-19) rapid test (Mologic Ltd.), using anterior nasal swab sampling (9, 10). The third, the Sure Status COVID-19 antigen card test (Premier Medical Corp. Pvt. Ltd.), using nasopharyngeal swab sampling, was approved by WHO (9). The reference standard was real-time (RT)-PCR using combined nasopharyngeal and oropharyngeal swab sampling. Study procedures were described previously (11). The study was conducted in collaboration with the Foundation for Innovative New Diagnostics (FIND), a WHO Collaborating Centre. Here, we report on an additional subanalysis regarding the B.1.1.7 lineage. This study was approved by the ethics committees of Charité - Universitätsmedizin (registration number EA1/371/20) and of the Heidelberg University Hospital (registration number S-180/2020).

Of 1,692 adults enrolled in the study, 354 (21%) tested positive by RT-PCR. Positive samples were typed for the N501Y and del69–70 polymorphisms by melting curve analysis. The presence of both polymorphisms was considered indicative of a B.1.1.7 lineage infection, an inference whose accuracy was confirmed by full-genome sequencing of all Heidelberg samples. For 22 patients (6%), typing was not done or was not possible due to the limit of detection of genotyping for samples with low viral loads (excluded from analysis). During the study, B.1.1.7 became the dominant SARS-CoV-2 lineage within only 5 weeks (12). Among the positive patients, 220 (62%) were infected with B.1.1.7, 3 (1%) with variant of concern (VOC) B.1.351, and 109 (31%) with other non-B.1.1.7 lineages. Three positive patients with viral typing results were excluded from the analysis due to invalid Ag-RDT results. Of the 329 positive patients included in the analysis, the majority (92.1%) were symptomatic. All three Ag-RDTs yielded comparable sensitivities, irrespective of an infection with the B.1.1.7 lineage (Table 1).

**TABLE 1:** Results of three Ag-RDTs for 329 RT-PCR-positive patients with viral typing results included in the analysis^*a*^

SARS-CoV-2 variant Ag-RDT	No. with positive Ag-RDT result	No. with negative Ag-RDT result	Sensitivity (95% CI) (%)	Duration of symptoms (median [IQR]) (days)	Viral load (median [IQR]) (log_10_ SARS-CoV2 RNA copies/ml)
Espline					
B.1.1.7	16	1	94.1 (73.0–99.7)	2 (1–3)	8.00 (7.36–8.58)
Others^*b*^	69	12	85.2 (75.9–91.3)	3 (1–4)	8.01 (6.52–8.82)
Sure Status					
B.1.1.7	71	5	93.4 (85.5–97.2)	3 (1–5)	8.22 (7.02–9.04)
Others^*b*^	17	2	89.5 (68.6–97.1)	2 (1–4)	8.11 (7.66–8.83)
Mologic^*c*^					
B.1.1.7	154	15	91.1 (85.9–94.5)	2 (1–4)	8.45 (7.51–9.17)
Others^*b*^	17	1	94.4 (74.2–99.7)	3 (2–4)	7.52 (6.88–8.40)

a Two Ag-RDTs (double testing) were used for 51 of the 329 patients, first with an anterior nasal swab sample (Mologic) and subsequently with a nasopharyngeal swab sample (Sure Status or Espline), leading to a total of 380 Ag-RDT results. Results from patients infected with B.1.1.7 are shown separately from those for other lineages. Sensitivities are overestimated due to the limit of detection of genotyping for samples with low viral loads. CI, confidence interval; IQR, interquartile range.

b Including 1 patient with VOC B.1.351 with positive Ag-RDT results (one with each test).

c Excluding 3 RT-PCR-positive patients with invalid Ag-RDT results.

Sensitivities are overestimated due to the limit of detection of genotyping for samples with low viral loads. Furthermore, the study is limited by the rapid emergence of the B.1.1.7 lineage during the course of the study, which led to an unequal distribution of this variant among the three Ag-RDTs. This did not allow a subanalysis by viral load, which could have potentially unmasked differences among the Ag-RDTs at low viral loads. Also, the study results are limited to mainly symptomatic patients.

There are only limited data on how N-gene mutations in VOCs may impact Ag-RDTs, and this study provides a clinical evaluation that complements analytical evaluations (6, 8, 13–16). To date, no major changes in test performance have been anticipated (7). However, test developers and health authorities should assess and monitor the impact of emerging variants on Ag-RDTs during development and postauthorization (17).

### Data availability.

All raw data and analysis code are available upon a request to the corresponding author.
